# Pulsatile Exophthalmos Due to Inadequate Orbital Reconstruction Following Fronto-Orbito-Ethmoidal Fibrous Dysplasia Resection

**DOI:** 10.7759/cureus.107055

**Published:** 2026-04-14

**Authors:** Joshua Estin, Roger Sheffmaker, Joseph Ifrach, Kevin Wojcik, Corey Mossop

**Affiliations:** 1 Department of Neurosurgery, Cooper Medical School of Rowan University, Camden, USA; 2 Department of Neurosurgery, Cooper University Hospital, Camden, USA; 3 Department of Neurosurgery, Medical College of Wisconsin, Milwaukee, USA

**Keywords:** case report, cranio-orbital surgery, fibrous dysplasia, iatrogenic complication, orbital reconstruction, pulsatile exophthalmos

## Abstract

Pulsatile exophthalmos can be a presenting sign of several orbital pathologies, such as trauma, iatrogenic injury, or congenital defect, or as a complication after orbital reconstructive surgery. Traditional approaches to management are conservative, with the goal being spontaneous resolution. The following case illustrates this rare but clinically significant complication and its treatment strategies.

A 48-year-old male underwent a bifrontal craniotomy with resection of a fibrous dysplasia, followed by reconstruction of the skull base and orbit with split-thickness calvarial autograft. Prior to leaving the operating room, the patient’s eye was pulsating, and the diagnosis of pulsatile exophthalmos was made. A discussion with the patient’s family resulted in the joint decision to undergo additional split-thickness calvarial bone grafting to further repair the lateral orbital wall. Postoperatively, the patient exhibited no further vision loss without evidence of CSF leak or additional complications.

This report presents a case of iatrogenic pulsatile exophthalmos with complete resolution of symptoms after additional reconstructive measures. This case serves to highlight the importance of the ocular examination prior to completion of cranial cases involving orbital reconstruction. Increased surveillance and timely correction of this finding may reduce the risk of disabling ocular symptoms.

## Introduction

Pulsatile exophthalmos is a clinically significant orbital finding associated with several underlying pathologies. Because of its varying etiologies, rarity, and its existence as a clinical sign rather than a disease entity, pulsatile exophthalmos’s incidence is not well-defined in the literature. Pulsatile exophthalmos most commonly arises from bony or vascular abnormalities that allow transmission of pulsatile intracranial or intraorbital forces into the globe [1]. This can be a result of trauma, as orbital roof fractures can disrupt the bony barrier, allowing direct pulsation from the dura or herniated brain tissue to be transmitted directly to the orbit [2]. Pulsatile exophthalmos can also be a result of congenital or pathological encephaloceles, such as a sphenorbital encephalocele of the anterior cranial fossa, as these can alter orbital volumes and introduce dynamic displacement to the globe [3]. Sphenoid wing dysplasia, as observed in conditions like neurofibromatosis type 1, is another well-described etiology of pulsatile exophthalmos due to malformation of the bony barrier and allowance of brain tissue to enter the orbital space [4,5]. Vascular causes such as carotid-cavernous fistula (CCF) can also produce pulsatile exophthalmos via increases in the vascular pressures within the orbital system [6].

Although orbital roof fractures, encephaloceles, sphenoid wing dysplasia, and CCFs are some of the most frequent etiologies, pulsatile exophthalmos has also been described as a rare iatrogenic complication of procedures that involve orbital reconstruction. In these cases, insufficient reconstruction of the orbit can result in transduction of intracranial pressure to the globe. Although there are no standardized management protocols for pulsatile exophthalmos, treatment strategies range from surgical intervention to observation with expectant spontaneous resolution [7-9]. However, persistent pulsatile exophthalmos can lead to substantial morbidity, including diplopia and vision loss if spontaneous resolution does not occur [10]. The potential for significant ophthalmologic morbidity warrants a nuanced discussion with patients and their loved ones immediately upon diagnosis regarding the options of watchful waiting versus additional surgical repair.

This case describes iatrogenic pulsatile exophthalmos following resection and reconstruction of fronto-orbito-ethmoidal fibrous dysplasia. This was discovered via a targeted intraoperative examination before anesthesia emergence, allowing for timely discussion with the patient’s family and immediate surgical intervention. Immediate and definite treatment was especially important for this patient, as he lives in an area with limited access to postoperative care. This case underscores the importance of meticulous orbital reconstruction, routine assessment for pulsatile exophthalmos in cases involving orbital reconstruction before leaving the operating room, and consideration of immediate surgical correction when reliable postoperative care may not be feasible.

## Case presentation

The patient is a 48-year-old male who presented with progressive facial deformity, pain, and several months of right-sided vision loss. On examination, he was alert and oriented to person, place, and time. Cranial nerves III through XII were intact. His examination was notable for a right inferior nasal visual field deficit, corroborated on formal visual field testing, and an ipsilateral inferolateral orbital dystopia. Motor strength was 5/5 in the bilateral upper and lower extremities. Sensation was intact to light touch and pinprick throughout. Reflexes were 2+ and symmetric bilaterally in the upper and lower extremities, with no Hoffman’s sign, Babinski reflex, or clonus. There was no pronator drift, and cerebellar testing was normal. Gait was unremarkable. Laboratory studies, including complete blood count, basic metabolic panel, and coagulation studies, were within normal limits.

**Figure 1:**
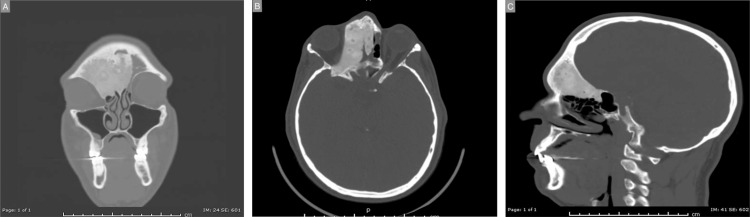
Preoperative CT. A: Coronal. B: Axial. C: Sagittal.

Computed tomography (CT) (Figure 1A-1C) and magnetic resonance imaging (MRI) revealed extensive fronto-orbito-ethmoidal fibrous dysplasia with compression of the right optic nerve.

**Figure 2:**
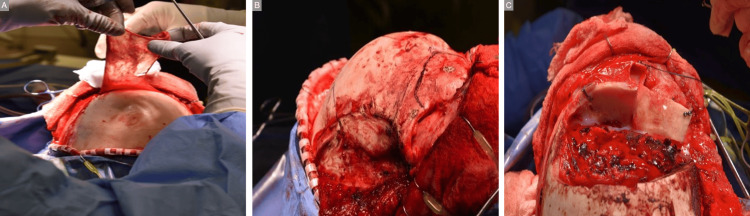
Initial operation. A: Harvesting of pericranial flap. B: Prior to resection of fibrous dysplasia. C: Original reconstruction using split-thickness calvarial autograft.

As a result, he underwent a bicoronal approach with harvesting of a rotational vascularized pericranial flap, bifrontal craniotomy, resection of the fibrous dysplasia with an extradural anterior clinoidectomy with optic nerve decompression, cranialization of the frontal and ethmoidal sinuses with abdominal fat and pericranial graft, and reconstruction of the anterior skull base and orbit with split-thickness calvarial autograft (Figure 2A-2C).

During the procedure, the eyes were draped out of the operative field in standard fashion, limiting direct assessment of the globe during reconstruction. Upon removing the sterile drapes and prior to leaving the operating room, the patient was found to have pulsatile exophthalmos (Video 1).

**Video 1:** Video of pulsatile exophthalmos.

**Figure 3:**
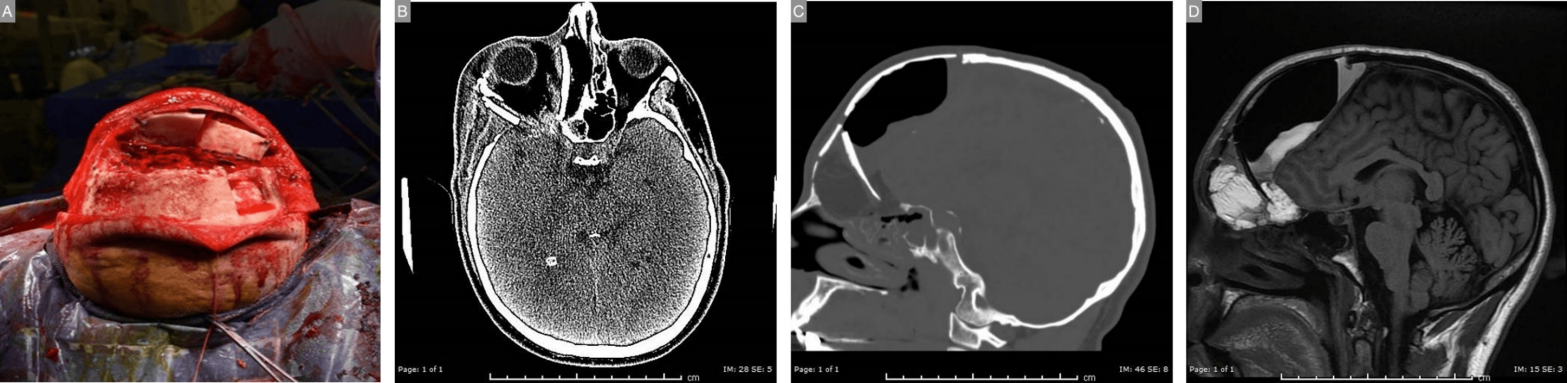
Revision and lateral reconstruction. A: Revision with additional split-thickness calvarial autograft. B-D: Postoperative imaging demonstrating reconstruction, reinforcement of the lateral orbit, and fat grafting.

The patient’s family was called and presented with the options of observation with hopes of spontaneous resolution versus immediate additional split-thickness calvarial bone graft harvesting and orbital reconstruction. After electing the latter option, the incision was reopened sterilely, and inadequate reconstruction of the lateral orbital wall was noted, which was repaired with additional split-thickness calvarial bone graft harvesting (Figure 3A). This resolved the pulsatile exophthalmos with postoperative CT and MRI’s showing expected post-surgical changes and expected pneumocephalus, which improved over time (Figure 3B-3D). The patient recovered well and displayed no new visual deficits without evidence of CSF leak or any additional complications at his three-month postoperative visit.

## Discussion

Pulsatile exophthalmos is an uncommon but clinically significant finding that can represent a broad spectrum of underlying pathologies. Well-recognized etiologies include congenital absence or dysplasia of the sphenoid wing, CCFs, orbital roof fractures, and arteriovenous malformations [6,11]. Iatrogenic causes must also be considered in the appropriate context. Traumatic causes warrant emphasis, as fractures of the orbit can allow for herniation of brain parenchyma into the orbit, transmitting pulsations and elevating intraorbital pressure. Mass effect may pose a direct risk to intraorbital structures, where the optic nerve is of particular vulnerability to ischemic or compressive injury [12,13]. In the absence of permanent visual sequelae, disabling symptoms such as persistent diplopia or ocular discomfort can profoundly affect patients’ quality of life [14,15]. 

Management of iatrogenic pulsatile exophthalmos requires careful consideration of the timing of presentation, severity of symptoms, and ability of the patient to follow up postoperatively. Medical and surgical treatment options include: clinical observation with hopes of spontaneous resolution, or additional surgical correction with split-thickness calvarial bone grafting, titanium mesh, or other allograft materials. Observation is a reasonable and conservative strategy in these patients, and several cases have documented spontaneous resolution [7,8,16]. Indications for surgical repair are more apparent intraoperatively or in the immediate perioperative or postoperative period, when the defect can be clearly visualized, and the opportunity exists for prompt reconstruction [2].

Reconstruction can be accomplished with several different materials. Split-thickness calvarial bone grafting offers the advantage of autologous material and long-term durability [9]. Alternatively, titanium mesh can provide a rigid scaffold that is well-suited for complex orbital defects [17,18]. Alloplastic implants, such as a porous polyethylene plate (Porex Corporation, Fairburn, GA, USA), represent another viable option, which can also be used in conjunction with titanium mesh for increased stability [19]. Regardless of the material used, reconstruction involves the creation of a robust barrier between the intracranial and orbital compartments.

Although rare, several cases of iatrogenic pulsatile exophthalmos have been reported in the literature. One notable report described the acute intraoperative onset of pulsatile exophthalmos in a patient with no history of NF1. Subsequent neurosurgical consultation and CT imaging revealed a previously unrecognized congenital absence of the sphenoid wing. In this particular case, surgical intervention was deferred, and the patient exhibited only low-grade pulsatile exophthalmos signs at follow-up [20]. Another case documented the development of transient postoperative pulsatile exophthalmos after craniotomy. This was characterized as “temporary” and resolved spontaneously without further intervention [16]. One study examining patients with sphenoid wing defects of mixed etiologies found that among this cohort, one patient experienced postoperative pulsatile exophthalmos that resolved spontaneously in one week [8].

This case adds to the limited body of literature on pulsatile exophthalmos by demonstrating that timely intraoperative recognition and prompt intervention can yield excellent outcomes. It highlights the value of proactive perioperative surveillance and the benefit of immediate correction when feasible, as it may prevent disabling morbidity and preserve long-term visual function.

## Conclusions

Pulsatile exophthalmos is an uncommon complication of orbital reconstruction, with most cases being identified postoperatively, having mild symptoms, and resolving spontaneously. This case demonstrates acute, intraoperative pulsatile exophthalmos discovered immediately following completion of a complex craniotomy involving major orbital reconstruction. Early recognition of abnormal globe pulsation after orbital reconstruction affords the opportunity to address the inadequate repair while still in the operating room. This spares the patient from the risks of pulsatile exophthalmos, reoperation, and irreversible damage to intraorbital structures. For this reason, intraoperative vigilance should be considered a critical safety step in orbital reconstructive surgery.

Moreover, management decisions must be individualized based on the patient’s access to postoperative care. In this case, the patient resided in the outer Hawaiian Islands, had limited access to specialty follow-up, and prioritized achieving definitive stability during the index operation. In resource-limited cases or cases with unlikely follow-up, proactive intraoperative correction of iatrogenic complications is often the most practical and safest course of action.
